# Gut bacteria interact directly with colonic mast cells in a humanized mouse model of IBS

**DOI:** 10.1080/19490976.2022.2105095

**Published:** 2022-07-29

**Authors:** Chiko Shimbori, Giada De Palma, Lauren Baerg, Jun Lu, Elena F. Verdu, David E. Reed, Stephen Vanner, Stephen M. Collins, Premysl Bercik

**Affiliations:** a Farncombe Family Digestive Health Research Institute, McMaster University, Hamilton, ON, Canada; b GIDRU, Queen's University, Kingston, ON, Canada

**Keywords:** Irritable bowel syndrome, bacteria, microbiota, mast cells, histamine 4 receptor, toll-like receptor 4

## Abstract

Both mast cells and microbiota play important roles in the pathogenesis of Irritable Bowel Syndrome (IBS), however the precise mechanisms are unknown. Using microbiota-humanized IBS mouse model, we show that colonic mast cells and mast cells co-localized with neurons were higher in mice colonized with IBS microbiota compared with those with healthy control (HC) microbiota. *In situ* hybridization showed presence of IBS, but not control microbiota, in the *lamina propria* and RNAscope demonstrated frequent co-localization of IBS bacteria and mast cells. TLR4 and H_4_ receptor expression was higher in mice with IBS microbiota, and in peritoneal-derived and bone marrow-derived mast cells (BMMCs) stimulated with IBS bacterial supernatant, which also increased BMMCs degranulation, chemotaxis, adherence and histamine release. While both TLR4 and H_4_ receptor inhibitors prevented BMMCs degranulation, only the latter attenuated their chemotaxis. We provide novel insights into the mechanisms, which contribute to gut dysfunction and visceral hypersensitivity in IBS.

## Introduction

The pathophysiology of Irritable Bowel Syndrome (IBS) is not fully elucidated, although low-grade gut inflammation, in particular mast cells, and altered gut microbiota have been implicated in its genesis.^1^ However, whether and how mast cell-gut microbiota interactions contribute to gut dysfunction in IBS is unknown.

IBS patients have higher numbers of colonic mast cells, often co-localized with enteric nerves, which was shown to correlate with the abdominal pain severity.^2,3^ Furthermore, increased mast cell degranulation was reported in colonic biopsies from IBS patients,^2,4,5^ suggestive of increased mast cell activation, that could modulate visceral sensitivity and epithelial barrier function through the release of neuroactive mediators.^2,3,6–8^ Indeed, mast cell stabilizers or histamine 1 receptor antagonist improved IBS symptoms and quality of life in clinical trials.^4,6,9^ The data thus suggest that mast cells, likely through production of histamine, could contribute to symptom generation in IBS, but the main driver for their migration into the gut and activation remains unclear.

Mouse studies have shown that commensal bacteria influence mast cell maturation, migration and tissue infiltration, and that mast cells play a key role in the control of bacterial infection.^10–12^ This interaction likely occurs through Toll-like receptor (TLR) 2 and 4 signaling.^10,13^ However, the precise pathways and bacterial metabolites involved in mast cell-microbiota communication are unknown.

Altered gut microbiota composition and metabolomic profiles have been described in IBS.^1^ We have previously shown that gut dysfunction and low-grade inflammation is transferred from IBS patients into mice by stool microbiota transplantation.^14^ Furthermore, our recent clinical study found that restriction of highly fermentable fiber improved symptoms in IBS patients, which was associated with changes in microbiota profiles and decreased urinary histamine, a main neuroactive metabolite of mast cells.^15^ Thus, in this study, we investigated whether gut microbiota could interact directly with mast cells using a variety of *in vitro* approaches and a validated microbiota-humanized mouse model of IBS.^14^

## Results and discussion

**Figure 1.**
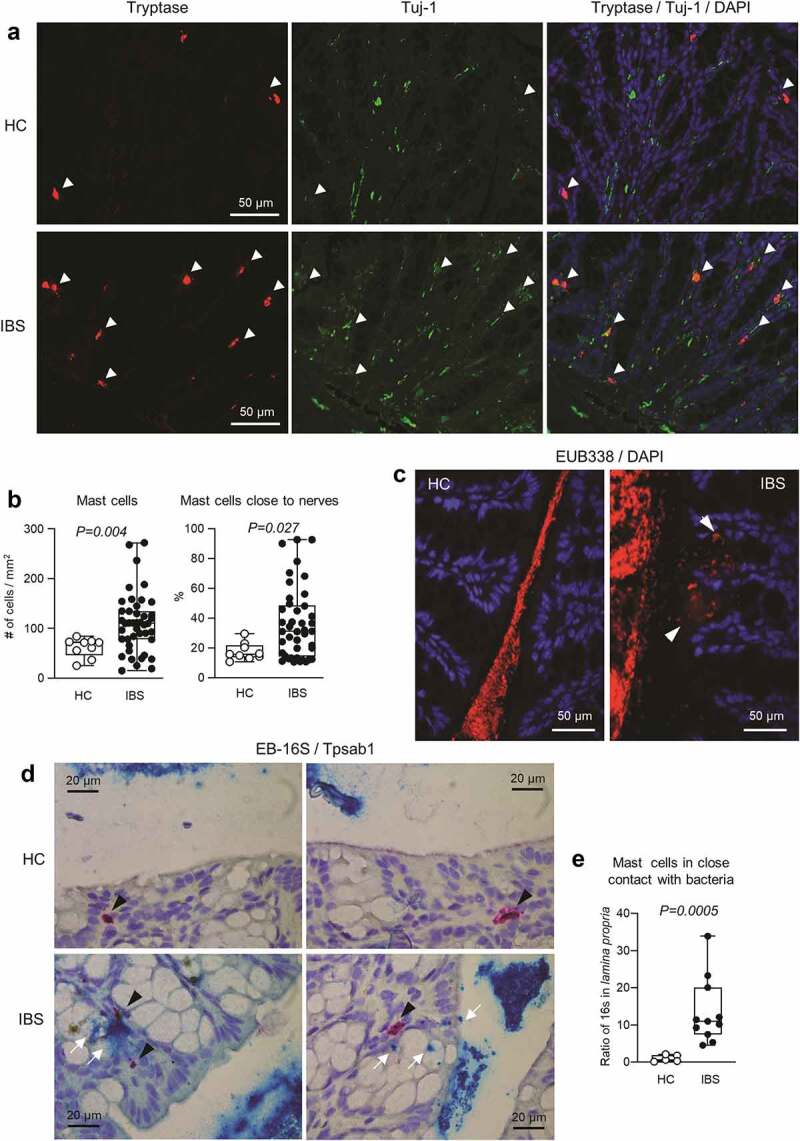
Colonic mast cells and gut bacteria in mice with human microbiota.

First, we colonized germ-free NIH Swiss mice with fecal microbiota from 2 selected IBS patients or 1 healthy control (at least 10 mice per human donor) and studied them three weeks later. We found that tryptase-immunoreactive mast cell counts were higher in the colon of mice colonized with IBS microbiota, compared with mice colonized with healthy control (HC) microbiota. Furthermore, most of the colonic mast cells in IBS mice were in close proximity (within 2 μm) to Tuj1-immunoreactive nerve fibers (Figure 1a,b). These results show that transfer of IBS microbiota induces higher colonic mast cell numbers in recipient mice, and that these mast cells are co-localized with enteric nerves, thus reproducing findings of clinical studies.^2,3^

**a**: Representative micrographs of immunofluorescent staining for mast cells-tryptase (red), neural fibers-Tuj-1 (green) in HC and IBS mice. **b**: Number of tryptase positive mast cells and number of mast cells in close proximity to nerves in HC (n = 9) and IBS (n = 42–44) mice. **c**: FISH staining of 16S rRNA (EUB-16S rRNA) in the colon of HC or IBS mice. Dapi in blue and EUB-16S-rRNA in red.**d**: Representative pictures of RNAscope staining of mast cells (tryptase, Tpsab1, red) and bacteria (16S rRNA, EUB-16SrRNA, blue). **e**: Quantification of RNAscope staining of mast cells and bacteria in HC (n = 5) and IBS (n = 11) mice. The data were analyzed with Mann–Whitney test (b, e).

Intestinal barrier function, which includes intact mucus layer, was reported to be altered in IBS,^1^ potentially allowing bacteria and their products to interact with the immune system. Direct bacterial interaction with mast cells was previously suggested,^16^ but never directly demonstrated. Our fluorescence *in situ* hybridization experiments showed a clear separation between gut bacteria and the colonic epithelium in HC mice, while in IBS mice gut bacteria were seen infiltrating the *lamina propria*, indicative of colonic mucus layer disruption (Figure 1c). Furthermore, RNAscope® CISH staining demonstrated that in IBS mice, but not HC mice, bacteria can be found in close proximity to colonic mast cells (Figure 1d), thus suggesting occurrence of direct bacteria-mast cell interactions. Although the exact mechanism underlying the intestinal barrier dysfunction observed in IBS patients is unknown, our data suggest that IBS microbiota disrupts the colonic mucus layer, enabling translocation of bacteria into *lamina propria*, with possible subsequent activation of mast cells and release of their mediators tryptase, chymase and histamine, which can further impair intestinal barrier function.^7,17–20^

Next, we analyzed the microbiota composition of IBS and HC mice. Microbial profiles differed between IBS and HC mice, with increased relative abundance of potentially pathogenic genera, such as *Escherichia-Shigella* spp. and *Eggerthella* spp., and a decrease in several potentially beneficial species such as *Akkermansia* spp. and members of the order Clostridiales in IBS mice (**Supplementary Figure S1A**). Furthermore, *Ruminococcus torques* group and *Coprobacillus* (Erysipelatoclostridiaceae) spp. relative abundance correlated with number of mast cells and mast cells co-localized with neural fibers, respectively (**Supplementary Figure S1B**).

**Figure 2.**
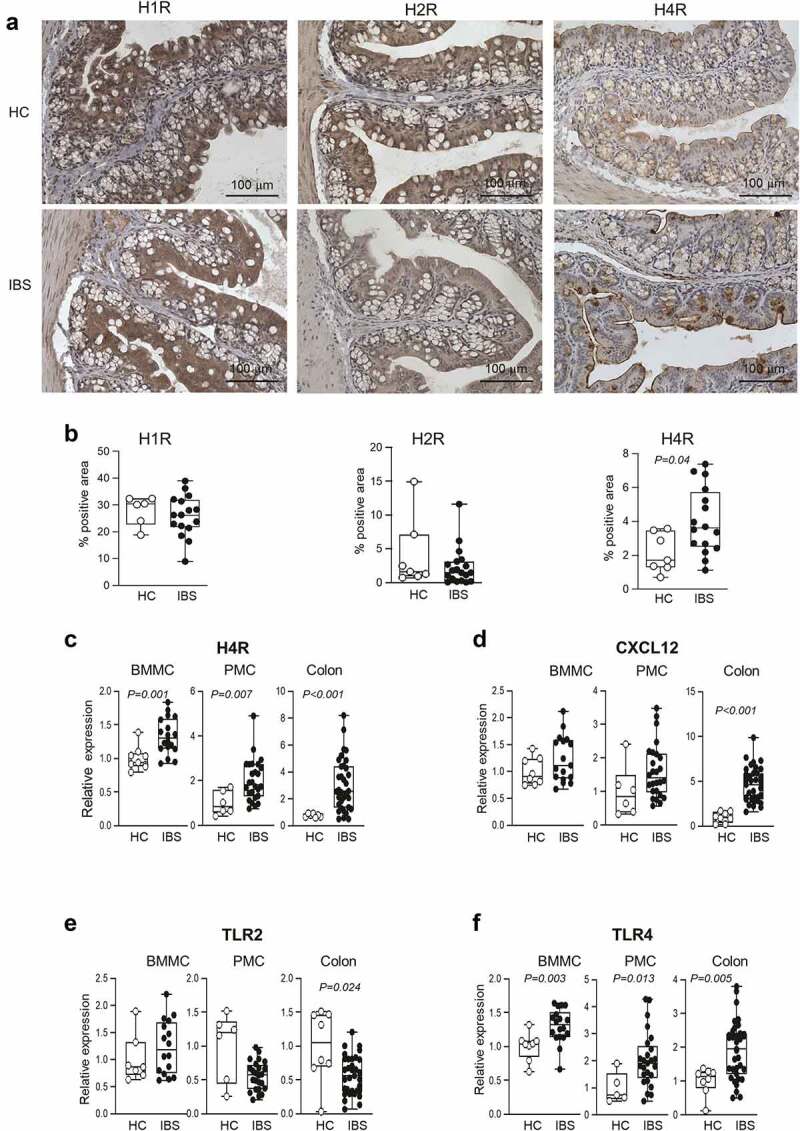
Higher colonic TLR4 and H_4_ receptor expression in IBS mice and mast cells incubated with IBS bacterial supernatant.

To investigate the putative mechanisms, by which IBS bacteria could communicate with mast cells, we studied levels of histamine receptors, TLRs and CXCL12, as these are established pathways of mast cell regulation. First, we assessed the expression of histamine receptors in colon tissues of microbiota-humanized by immunohistochemistry. We found that while H_1_ and H_2_ receptor levels were similar between IBS and HC mice, H_4_ receptor expression was higher in IBS mice (Figure 2a,b). Similar results were obtained when we analyzed RNA gene expression in colon tissues of microbiota-humanized mice, or in bone marrow-derived mast cells (BMMCs) and peritoneal-derived mast cells (PMCs) incubated with supernatants of bacterial cultures from IBS and HC mice. These experiments showed consistently elevated H_4_ receptor expression in colonic tissues, BMMCs and PMCs (Figure 2c). Histamine, produced by both mast cells and bacteria,^21^ plays an important role in gut function, including motility and visceral sensitivity.^22,23^ Although mast cells express H_1_, H_2_ and H_4_ receptors,^22^ H_4_ receptor has the highest affinity to histamine, and plays an important role in mast cell migration and visceral hypersensitivity.^24,25^

**a**: Representative micrographs of H_1_,H_2_, and H_4_ receptor immunoreactivity (brown) in the colon of HC and IBS mice. **b**: Quantification of H_1_,H_2_, and H_4_ receptor immunoreactivity in the colon of HC (n = 7) and IBS (n = 16) mice. **c, d**: H_4_ receptor and CXCL12 mRNA measured by qPCR in colon tissues, or BMMCs and PMCs co-cultured with IBS or HC microbial culture supernatants. All data were normalized against gapdh and β-actin. BMMC: HC (n = 8–9), IBS (n = 17–18). PMC: HC (n = 5–6), IBS (n = 24–26). Colon tissues: HC (n = 7–8), IBS (n = 32–35). **e, f**: TLR2 and TLR4 mRNA measured by qPCR in BMMC, PMC, and colon tissues. BMMCs and PMCs were co-cultured with IBS or HC microbial culture supernatants. All data were normalized against gapdh and β-actin. BMMC: HC (n = 8–9), IBS (n = 17–18). PMC: HC (n = 5–6), IBS (n = 24–26). Colon tissues: HC (n = 7–8), IBS (n = 32–35). The data were analyzed with Mann–Whitney test (b, c, d, e, f).

CXCL12 is a potent chemoattractant and activator of mast cells.^25^ Although its expression was higher in colonic tissues of IBS mice (Figure 2d), it was similar in BMMCs and PMCs (Figure 2d), suggesting it may contribute, but it is likely not a key player in the communication between IBS microbiota and mast cells.

We then assessed expression of TLR2 and TLR4 (Figure 2e,f), as they were previously implicated in microbiota-mast cell interactions. TLR2 expression was similar in BMMCs and PMCs incubated with bacterial supernatants from IBS or HC mice, and lower in colonic tissues of IBS mice. In contrast, TLR4 expression was consistently higher in BMMCs, PMCs and colonic tissues from IBS mice. These data are in agreement with several clinical studies showing a higher TLR4 mRNA and protein levels in the colonic mucosa of IBS patients,^26–28^ suggesting that TLR4 pathway is involved in pathogenesis of IBS.

**Figure 3.**
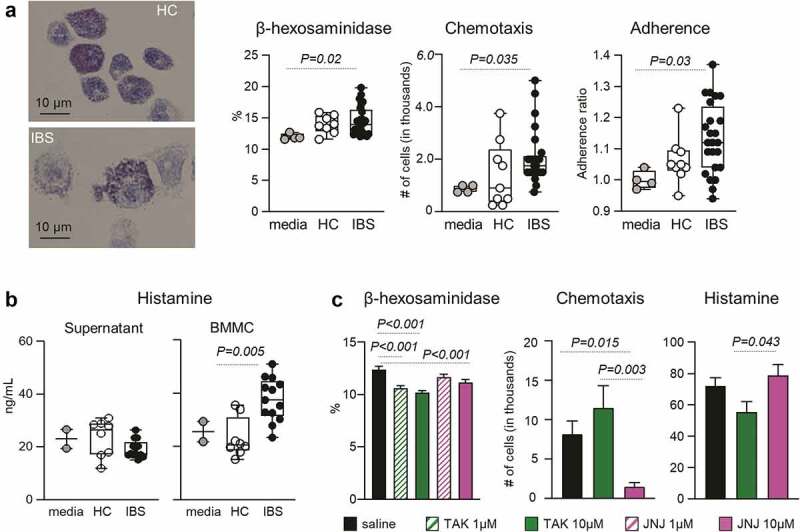
H_4_ receptor and TLR4 pathways mediate mast cell degranulation, activation and chemotaxis.

Next, we investigated the effect of gut microbiota on mast cell functions using BMMCs co-cultured with bacterial cultures’ supernatants from IBS and HC mice. BMMCs degranulation, adherence and chemotaxis increased when incubated with IBS supernatant compared to control media (Figure 3a). Furthermore, IBS but not HC supernatant stimulated histamine release from BMMCs (Figure 3b), altogether indicating that microbial products contained in IBS culture supernatants can induce migration and activation of mast cells.

**a**: Representative micrographs of Toluidine Blue staining of BMMCs co-cultured with IBS or HC microbial supernatants. Mast cell degranulation was assessed by β-hexosaminidase assay. Medium (n = 4), HC (n = 9), IBS (n = 24–25). **b**: Histamine produced by the microbiota and by mast cells. Histamine levels in the microbial supernatants (left) and BMMC co-cultured with microbial supernatants (right) were measured by ELISA. Medium (n = 2), HC (n = 8), IBS (n = 13). **c**: Role of TLR4 and H_4_ receptor pathways in mast cell function. Degranulation, chemotaxis and histamine release by BMMC incubated with microbial supernatants, with and without H_4_R antagonist JNJ-7777120 (JNJ) or TLR4 inhibitor TAK-242 (TAK). Degranulation n = 30, histamine release n = 14, and chemotaxis n = 9 in each group, respectively. The data were analyzed by Kruskal–Wallis test followed by Dunn’s post-hoc test (a, b, c).

To discern the respective roles of H_4_ receptor and TLR4 pathways in bacterial-mast cells interactions, we pharmacologically blocked H_4_ and TLR4 receptors. Both H_4_ receptor antagonist JNJ-7777120 (JNJ) and TLR4 inhibitor TAK-242 (TAK) attenuated IBS supernatant-induced BMMCs degranulation, but only JNJ inhibited BMMCs chemotaxis (Figure 3c). Furthermore, TAK-treated BMMCs released less histamine than JNJ-treated BMMCs (Figure 3c). Thus, these experiments indicate that activation of both TLR4 and H_4_ receptors leads to mast cell degranulation, but only H_4_ receptor pathway is critical for mast cell migration while TLR4 modulates histamine production.

Overall, we provide novel insights into the mechanisms behind the attraction and activation of mast cells mediated by TLR4 and H_4_ receptor mediated pathways, including the possibility that gut bacteria engage in direct contact with intestinal mast cells, contributing to the gut dysfunction and visceral hypersensitivity observed in IBS.

## Methods

*(For additional details, please see the Supplemental Methods)*

### Animals

Germ-free NIH Swiss mice (8–10 weeks old) from the Axenic Gnotobiotic Unit of McMaster University were gavaged with diluted human fecal samples and housed for three weeks in sterilized racks, as previously described.^14^ All experiments were approved by the McMaster University Animal Care Committee under the Animal Utilization Protocol #18-08-35.

### Fecal microbiota analysis

Total genomic DNA was extracted from the cecal samples, V3 region of the 16S rRNA gene amplified, and Illumina sequencing performed as previously described.^29,30^

### Immunofluorescence

Immunofluorescence on formalin-fixed tissues was performed using mast cell tryptase (ab151757 Rabbit, Abcam), Tuj-1 (ab78078 Mouse, Abcam) as primary antibodies; and Alexa Fluor® 555-conjugated donkey anti-rabbit (A-31572, Invitrogen) and Alexa Fluor® 488-conjugated donkey anti-Guinea Pig (706–545-148, Jackson ImmunoResearch) as secondary antibodies.

### Fluorescence in situ hybridization (FISH) and RNA scope®

FISH was performed on Carnoy’s solution-fixed paraffin-embedded section, using Cy3 conjugated EUB338 probe (Integrated DNA Technologies).

RNA chromogenic *in situ* hybridization (CISH) was performed on formalin-fixed sections using the RNAscope® 2.5 LS Duplex Reagent kit (322440), EUB-16S-rRNA (464468) and Mm-Tpsab1-C2 (432948-C2, Advanced Cell Diagnostics).

### Bone marrow mast cell (BMMC) and peritoneal-derived mast cells (PMCs)

BMMC and PMCs were obtained from healthy mice as described previously.^31,32^

### Bacteria supernatant and BMMC co-culture

Diluted cecal samples were inoculated into semi-defined medium, LDMIII69 for 20 hours, then centrifuged, sterile-filtered and used for co-culture with BMMC for 4 hours.

### β-Hexosaminidase release

Degranulation studies were performed by measuring β-hexosaminidase release, as described previously.^31^

### Chemotaxis assay

Chemotaxis studies were performed using Transwell® Permeable Supports with 8.0 μm pore polycarbonate membrane on 6.5 mm inserts in 24-well polystyrene plates, as described previously.^33^

### Colonic and BMMC gene expression

BMMC (5.6 × 10^6^ cells/mL), co-cultured with 10% bacterial supernatant for 4 hours at 37°C, were collected and centrifuged. Freshly collected colon tissues were stored in RNAlater (Sigma) at −80°C. Cell pellets and colon tissues were dissolved or homogenized with RLT buffer (Qiagen, Toronto, Canada) containing 1% β-mercaptoethanol. Total RNA extractions were conducted with the RNeasy Mini Kit (Qiagen, Toronto, Canada) according to the manufacturer’s instructions. The mRNA expression of mouse intestinal H_4_R, TLR2, TLR4, and CXCL12 were analyzed by real-time qPCR using a CFX Connect Real-Time System (Bio-Rad and Applied Biosystems).

### Histamine ELISA

Histamine was measured with the Mouse Histamine ELISA Kit (LS-F28398, LifeSpan Biosciences, Burlington, Canada), according to manufacturer’s instructions.

### Statistical analysis

The data are presented as median (IQD) or mean ± SEM. The data were analyzed by Kruskal-Wallis test followed by Dunn’s posttest or Mann-Whitney test. Associations between tryptase positive cells and microbial genera were analyzed with the Spearman’s rank correlation test. The resulting P values were corrected for multiple comparisons, allowing 5% of False Discovery Rate. P < .05 was considered statistically significant.

## Supplementary Material

Supplemental Material

## Data Availability

The data contained this study are available at https://drop.mcmaster.ca/s/9Ktqsmkn3K2Pcdd.

## References

[1] Collins SM. A role for the gut microbiota in IBS. Nat Rev Gastroenterol Hepatol. 2014;11:497–9. doi:10.1038/nrgastro.2014.40. 24751910

[2] Barbara G, Stanghellini V, De Giorgio R, Cremon C, Cottrell GS, Santini D, Pasquinelli G, Morselli-Labate AM, Grady EF, Bunnett NW, et al. Activated mast cells in proximity to colonic nerves correlate with abdominal pain in irritable bowel syndrome. Gastroenterology. 2004;126(3):693–702. doi:10.1053/j.gastro.2003.11.055. 14988823

[3] Bashashati M, Moossavi S, Cremon C, Barbaro MR, Moraveji S, Talmon G. Colonic immune cells in irritable bowel syndrome: a systematic review and meta-analysis. Neurogastroenterol Motil. 2018;30(1). doi:10.1111/nmo.13192. 28851005

[4] Lobo B, Ramos L, Martínez C, Guilarte M, González-Castro AM, Alonso-Cotoner C, Pigrau M, Torres I, Rodiño‐Janeiro BK, Salvo‐Romero E, et al. Downregulation of mucosal mast cell activation and immune response in diarrhoea-irritable bowel syndrome by oral disodium cromoglycate: a pilot study. United European Gastroenterol J. 2017;5:887–897. doi:10.1177/2050640617691690. PMC5625876 29026603

[5] Liu DR, Xu XJ, Yao SK. Increased intestinal mucosal leptin levels in patients with diarrhea-predominant irritable bowel syndrome. World J Gastroenterol. 2018;24:46–57. doi:10.3748/wjg.v24.i1.46. 29358881 PMC5757124

[6] Klooker TK, Braak B, Koopman KE, Welting O, Wouters MM, van der Heide S, Schemann M, Bischoff SC, van den Wijngaard RM, Boeckxstaens GE, et al. The mast cell stabiliser ketotifen decreases visceral hypersensitivity and improves intestinal symptoms in patients with irritable bowel syndrome. Gut. 2010;59:1213–1221. doi:10.1136/gut.2010.213108. 20650926

[7] Martínez C, Vicario M, Ramos L, Lobo B, Mosquera JL, Alonso C, Sánchez A, Guilarte M, Antolín M, de Torres I, et al. The jejunum of diarrhea-predominant irritable bowel syndrome shows molecular alterations in the tight junction signaling pathway that are associated with mucosal pathobiology and clinical manifestations. Am J Gastroenterol. 2012;107(5):736–746. doi:10.1038/ajg.2011.472. 22415197

[8] Vanuytsel T, van Wanrooy S, Vanheel H, Vanormelingen C, Verschueren S, Houben E, Salim Rasoel S, Tόth J, Holvoet L, Farré R, et al. Psychological stress and corticotropin-releasing hormone increase intestinal permeability in humans by a mast cell-dependent mechanism. Gut. 2014;63(8):1293–1299. doi:10.1136/gutjnl-2013-305690. 24153250

[9] Wouters MM, Balemans D, Van Wanrooy S, Dooley J, Cibert-Goton V, Alpizar YA, Valdez-Morales EE, Nasser Y, Van Veldhoven PP, Vanbrabant W, et al. Histamine receptor H1-mediated sensitization of TRPV1 mediates visceral hypersensitivity and symptoms in patients with irritable bowel syndrome. Gastroenterology. 2016;150:875–87.e9. doi:10.1053/j.gastro.2015.12.034. 26752109

[10] Kunii J, Takahashi K, Kasakura K, Tsuda M, Nakano K, Hosono A, Kaminogawa S. Commensal bacteria promote migration of mast cells into the intestine. Immunobiology. 2011;216:692–697. doi:10.1016/j.imbio.2010.10.007. 21281976

[11] Schwarzer M, Hermanova P, Srutkova D, Golias J, Hudcovic T, Zwicker C, Sinkora M, Akgün J, Wiedermann U, Tuckova L, et al. Germ-Free mice exhibit mast cells with impaired functionality and gut homing and do not develop food allergy. Front Immunol. 2019;10:205. doi:10.3389/fimmu.2019.00205. 30809227 PMC6379318

[12] Wang Z, Mascarenhas N, Eckmann L, Miyamoto Y, Sun X, Kawakami T, Di Nardo A. Skin microbiome promotes mast cell maturation by triggering stem cell factor production in keratinocytes. J Allergy Clin Immunol. 2017;139(4):1205–16.e6. doi:10.1016/j.jaci.2016.09.019. 27746235 PMC5385284

[13] Sandig H, Bulfone-Paus S. TLR signaling in mast cells: common and unique features. Front Immunol. 2012;3:185. doi:10.3389/fimmu.2012.00185. 22783258 PMC3389341

[14] De Palma G, Lynch MD, Lu J, Dang VT, Deng Y, Jury J, Umeh G, Miranda PM, Pigrau Pastor M, Sidani S, et al. Transplantation of fecal microbiota from patients with irritable bowel syndrome alters gut function and behavior in recipient mice. Sci Transl Med. 2017;9(379). doi:10.1126/scitranslmed.aaf6397. 28251905

[15] McIntosh K, Reed DE, Schneider T, Dang F, Keshteli AH, De Palma G, Madsen K, Bercik P, Vanner S. FODMAPs alter symptoms and the metabolome of patients with IBS: a randomised controlled trial. Gut. 2017;66(7):1241–1251. doi:10.1136/gutjnl-2015-311339. 26976734

[16] Zhang L, Song J, Hou X. Mast cells and irritable bowel syndrome: from the bench to the bedside. J Neurogastroenterol Motil. 2016;22(2):181–192. doi:10.5056/jnm15137. 26755686 PMC4819856

[17] Martínez C, Lobo B, Pigrau M, Ramos L, González-Castro AM, Alonso C, Guilarte M, Guilá M, de Torres I, Azpiroz F, et al. Diarrhoea-predominant irritable bowel syndrome: an organic disorder with structural abnormalities in the jejunal epithelial barrier. Gut. 2013;62(8):1160–1168. doi:10.1136/gutjnl-2012-302093. 22637702

[18] Fu Z, Thorpe M, Hellman L, Boudinot P. rMCP-2, the major rat mucosal mast cell protease, an analysis of its extended cleavage specificity and its potential role in regulating intestinal permeability by the cleavage of cell adhesion and junction proteins. PLoS One. 2015;10(6):e0131720. doi:10.1371/journal.pone.0131720. 26114959 PMC4482586

[19] Groschwitz KR, Wu D, Osterfeld H, Ahrens R, Hogan SP. Chymase-mediated intestinal epithelial permeability is regulated by a protease-activating receptor/matrix metalloproteinase-2-dependent mechanism. Am J Physiol Gastrointest Liver Physiol. 2013;304(5):G479–89. doi:10.1152/ajpgi.00186.2012. 23306080 PMC3602679

[20] Piche T, Barbara G, Aubert P, Bruley Des Varannes S, Dainese R, Nano JL, Cremon C, Stanghellini V, De Giorgio R, Galmiche JP, et al. Impaired intestinal barrier integrity in the colon of patients with irritable bowel syndrome: involvement of soluble mediators. Gut. 2009;58(2):196–201. doi:10.1136/gut.2007.140806. 18824556

[21] Branco ACCC, Yoshikawa FSY, Pietrobon AJ, Sato MN. Role of histamine in modulating the immune response and inflammation. Mediators Inflamm. 2018;2018:9524075. doi:10.1155/2018/9524075. 30224900 PMC6129797

[22] Thangam EB, Jemima EA, Singh H, Baig MS, Khan M, Mathias CB, Church MK, Saluja R. The role of histamine and histamine receptors in mast cell-mediated allergy and inflammation: the hunt for new therapeutic targets. Front Immunol. 2018;9:1873. doi:10.3389/fimmu.2018.01873. 30150993 PMC6099187

[23] Fargeas MJ, Fioramonti J, Bueno L. Involvement of different receptors in the central and peripheral effects of histamine on intestinal motility in the rat. J Pharm Pharmacol. 1989;41:534–540. doi:10.1111/j.2042-7158.1989.tb06521.x. 2571697

[24] Deiteren A, De Man JG, Pelckmans PA, De Winter BY. Histamine H₄ receptors in the gastrointestinal tract. Br J Pharmacol. 2015;172:1165–1178. doi:10.1111/bph.12989. 25363289 PMC4337694

[25] Godot V, Arock M, Garcia G, Capel F, Flys C, Dy M, Emilie D, Humbert M. H4 histamine receptor mediates optimal migration of mast cell precursors to CXCL12. J Allergy Clin Immunol. 2007;120(4):827–834. doi:10.1016/j.jaci.2007.05.046. 17681365

[26] Brint EK, MacSharry J, Fanning A, Shanahan F, Quigley EM. Differential expression of toll-like receptors in patients with irritable bowel syndrome. Am J Gastroenterol. 2011;106(2):329–336. doi:10.1038/ajg.2010.438. 21102570

[27] Belmonte L, Beutheu Youmba S, Bertiaux-Vandaële N, Antonietti M, Lecleire S, Zalar A, Gourcerol G, Leroi A-M, Déchelotte P, Coëffier M, et al. Role of toll like receptors in irritable bowel syndrome: differential mucosal immune activation according to the disease subtype. PLoS One. 2012;7(8):e42777. doi:10.1371/journal.pone.0042777. 23028414 PMC3461726

[28] Jalanka J, Lam C, Bennett A, Hartikainen A, Crispie F, Finnegan LA, Cotter PD, Spiller R. Colonic gene expression and fecal microbiota in diarrhea-predominant irritable bowel syndrome: increased toll-like receptor 4 but minimal inflammation and no response to mesalazine. J Neurogastroenterol Motil. 2021;27(2):279–291. doi:10.5056/jnm20205. 33795545 PMC8026366

[29] Bartram AK, Lynch MD, Stearns JC, Moreno-Hagelsieb G, Neufeld JD. Generation of multimillion-sequence 16S rRNA gene libraries from complex microbial communities by assembling paired-end illumina reads. Appl Environ Microbiol. 2011;77(11):3846–3852. doi:10.1128/AEM.02772-10. 21460107 PMC3127616

[30] Whelan FJ, Surette MG. A comprehensive evaluation of the sl1p pipeline for 16S rRNA gene sequencing analysis. Microbiome. 2017;5(1):100. doi:10.1186/s40168-017-0314-2. 28807046 PMC5557527

[31] Shimbori C, Upagupta C, Bellaye PS, Ayaub EA, Sato S, Yanagihara T, Zhou Q, Ognjanovic A, Ask K, Gauldie J, et al. Mechanical stress-induced mast cell degranulation activates TGF-beta1 signalling pathway in pulmonary fibrosis. Thorax. 2019;74:455–465. doi:10.1136/thoraxjnl-2018-211516. 30808717

[32] Larson D, Mitre E. Histamine release and surface CD200R1 staining as sensitive methods for assessing murine mast cell activation. J Immunol Methods. 2012;379(1–2):15–22. doi:10.1016/j.jim.2012.02.014. 22394590 PMC3328686

[33] Khambati I, Han S, Pijnenburg D, Jang H, Forsythe P. The bacterial quorum-sensing molecule, N-3-oxo-dodecanoyl-L-homoserine lactone, inhibits mediator release and chemotaxis of murine mast cells. Inflamm Res. 2017;66(3):259–268. doi:10.1007/s00011-016-1013-3. 27896412

