# Supplementary material for: Gut bacteria interact directly with colonic mast cells in a humanized mouse model of IBS
Source: Gut Microbes. 2022 Jul 29;14(1):2105095. doi: 10.1080/19490976.2022.2105095 (PMC9341375; doi:10.1080/19490976.2022.2105095)
Supplement: Supplemental Material [file KGMI_A_2105095_SM9816.docx]

*Supplemental online material*

**Gut bacteria interact directly with colonic mast cells in a humanized mouse model of IBS**

Chiko Shimbori^1*^, Giada De Palma^1*^, Lauren Baerg^1^, Elena F. Verdu^1^, David E. Reed^2^, Stephen Vanner^2^, Stephen M. Collins^1^, Premysl Bercik^1#^

^1^Farncombe Family Digestive Health Research Institute, McMaster University

^2^GIDRU, Queens University

^*^ These first authors contributed equally to this work.

^#^Corresponding author. Email: bercikp@mcmaster.ca

**Material and Method**

*Animals*

Germ-free NIH Swiss mice (8-10 weeks old) were obtained from the Axenic Gnotobiotic Unit of McMaster University and colonized with fecal microbiota from two IBS patients (non-constipation), and one healthy control (HC). The mice were gavaged with diluted human fecal samples and housed for three weeks in sterilized individually ventilated racks, on a 12h:12h light-dark cycle with free access to food and water, as previously described.^1^ Mice were sacrificed in a standardized manner using Isoflurane (Fresenius Kabi Canada, Toronto, ON) anaesthesia. All experiments were approved by the McMaster University Animal Care Committee under the Animal Utilization Protocol #18-08-35.

*Fecal microbiota analysis*

Stool samples were collected 3 weeks after colonization and immediately frozen at -80°C. Colonic, and cecal content samples were collected at sacrifice. Total genomic DNA was extracted from the cecal samples as previously described.^2^ Following this protocol, amplification of the V3 region of the 16S rRNA gene, and Illumina sequencing were performed as previously described.^2, 3^ Briefly, the data was analyzed following the pipelines of dada2^4^ and QIIME2.^5^ Taxonomic assignments were performed using the RDP classifier^6^ with the Silva small subunit Ref. NR99 138.1 database^7, 8^ (2020) training set. Analyses were done using either QIIME2,^5^ MaAsLin,^9^ and Phyloseq package (1.28)^10^ for R (3.6.1), and SPSS software v. 23. Prior to taxonomic analyses (ANCOM and MaAsLin), the dataset was filtered, eliminating Amplicon sequence variants (ASVs) with a frequency < 10, that were not present at least in 5 samples. The data was either transformed into relative abundance or rarefied at the maximum minimum common cut-off (32,970), and the results were comparable. All results were corrected for multiple comparisons, allowing 5% of False Discovery Rate.

*Immunofluorescence*

For tryptase and Tuj-1 staining, tissues were fixed overnight in 10% formalin, and sections were paraffin embedded and mounted on microscope slides. Following deparaffinization, slides were incubated with 0.1% Sudan Black B solution to reduce autofluorescence, steamed with citric acid buffer for antigen retrieval, and non-specific sites were saturated with 5% BSA in PBS. Sections were then incubated at 4°C overnight with primary antibodies; mast cell tryptase (ab151757 Rabbit, Abcam ,ON, Canada) diluted 1:200, and Tuj-1 (ab78078 Mouse, Abcam, ON, Canada) diluted 1:100. Conjugated secondary antibodies; Alexa Fluor® 555-conjugated donkey anti-rabbit (A-31572, Invitrogen) and Alexa Fluor® 488-conjugated donkey anti-Guinea Pig (706-545-148, Jackson ImmunoResearch Inc. PA, USA), were applied to sections and incubated for 1 hour at room temperature. All antibodies were diluted in 1% BSA in PBS. Slides were mounted in ProLong-Gold with DAPI (ProLong® Gold antifade regent with DAPI; Thermo Fisher Scientific, ON, Canada). Pictures were taken using an epifluorescence microscope (Eclipse 80: Nikon, ON, Canada) with the same setting and exposure time for all pictures. For each colon section, 8-12 micrographs were taken from mucosa and submucosa. The quantification was performed with FIJI/Image J.

*Fluorescence In Situ Hybridization and RNA scope*®

Fluorescent *in situ* hybridization (FISH) was performed on colon sections that were fixed with Carnoy’s solution and paraffin embedded. Following deparaffinization, non-specific sites were blocked using 2% BSA in hybridization buffer and the slides were incubated with Cy3 conjugated EUB338 probe (Integrated DNA Technologies, Coralville, IA), diluted 1:10 in hybridization buffer, at 50°C overnight. Slides were mounted in ProLong-Gold Antifade with DAPI (Thermo Fisher Scientific, ON, Canada). Images were captured using an epifluorescence microscope (Eclipse 80: Nikon, ON, Canada) with the same setting and exposure time for all pictures.

RNA chromogenic in situ hybridization (CISH) was performed on colon sections that were 10% formalin fixed overnight, paraffin embedded and stained at the Core Histology Facility at McMaster University. RNA CISH assays were performed using the RNAscope® 2.5 LS Duplex Reagent kit (322440, Advanced Cell Diagnostics (ADC), Newark, CA), according to the manufacturer’s instructions. Staining was performed on the Leica Bond RX automated stainer, using a program devised by ACD for the Leica Bond. Slides were incubated with the RNAscope® 2.5 LS -ISH probes, EB-16S-rRNA (464468, ADC) and Mm-Tpsab1-C2 (432948-C2, ADC), for 2 hours at 40°C. Slides were then visualized with a red chromogen from the Bond Polymer Refine Red Detection kit (DS9390, Leica Biosystems) and a green chromogen RNAscope® 2.5 LS Green Accessory Pack (322550, ADC) before mounting slides with VectaMount Permanent (H-5000, Vector Labs, Burlington, Canada). Images were captured using Nikon microscope (Eclipse 80: Nikon, ON, Canada).

*Bone Marrow mast cell*

Mouse bone marrow derived mast cells (BMMC) were obtained from additional healthy mice as described previously.^11^ Briefly, bone marrow cells were cultured for up to 10 weeks in enriched RPMI-1640 medium in the presence of 50 ng/ml rmSCF plus 10 ng/ml rmIL-3 (R&D Systems, Minneapolis, MN). Non-adherent cells were hemidepleted twice each week with enriched medium. Cells were used for the experiments after 4-8 weeks of culture. After 4 weeks of culture, at which point these primary populations were 90% mast cells, based on staining toluidine blue staining and staining for c-Kit and FcεRI expression (Biolegend, San Diego, CA).

*Cytospin and Toluidine Blue staining*

BMMC (5 x 10^4^ cells/slide) were cytocentrifuged onto cytospin slides, using the Shandon Cytospin 4 Cytocentrifuge (A78300003, Thermo Fisher Scientific, ON, Canada), following a standard procedure. In brief, slides were fixed using Mota’s fixative, and stained with acid toluidine blue solution for 15 minutes at room temperature. Slides were mounted with Paramount and images were captured by Nikon microscope (Eclipse 80: Nikon, ON, Canada).

C*ecal bacteria supernatant and mouse bone marrow derived mast cells (BMMC) co-culture*

Mouse cecal samples were diluted 1:10 (weight : volume) with sterile PBS and 10 μL were inoculated into 5 mL of semi-defined medium, LDMIII69. After 20 hours, cultures were centrifuged and supernatants were collected, filtered with 0.22 μm-sized syringe filters (Millipore, Etobicoke, Canada), and stored at -80°C until analyses. Mouse bone marrow derived mast cells (BMMC) were obtained as described above, and co-cultured with 10% cultures’ supernatant for 4 hours.

*β-Hexosaminidase release*

Degranulation studies were performed by measuring b-hexosaminidase release, as described previously.^11^ Briefly, mast cells were starved of antibiotics and growth factors overnight, washed, and resuspended in 0.04% BSA HEPES buffer (5x10^4^ / 100 μL). For inhibitor assays, BMMC were preincubated for 1 hour at 37°C with or without inhibitors; histamine H_4_R antagonist JNJ-7777120 (1 μM, 10 μM: ab144405, Abcam, ON, Canada) or TLR4 inhibitor TAK-242 (1 μM, 10 μM: 13871, Cayman Chemical, Burlington, Canada). BMMC were transferred to 96-well polystyrene tissue culture plates, and the cells were incubated with 10% cecal bacteria supernatant for 4 hours at 37°C. Reactions were stopped by centrifuging the cells (900 rpm, 5 min), and 50 μl of supernatant was mixed with 100 μl of b-hexosaminidase substrate (4-nitrophenyl N-acetylb-D-glucosaminide) solution adjusted to pH = 4.5 with citrate buffer. After incubating for 2 h at 37 °C, 50 μl of 0.4 M glycine solution (pH = 10.7) was added, and the absorbance at 405 nm was measured. Total release of mast cell contents was achieved by adding 1% Triton X-100 to the remaining cell pellet and carrying out the same procedure as outlined above. Degranulation percentage was calculated using the formula: (b-Hex(supernatant)-A)/(b-Hex(total)-A) x 100, where A is the b-hexosaminidase released from unstimulated cells.

*Chemotaxis Assay*

Chemotaxis studies were performed using Transwell^®^ Permeable Supports with 8.0 μm pore polycarbonate membrane on 6.5 mm inserts in 24-well polystyrene plates, as described previously.^12^ BMMC were starved of antibiotics and growth factors overnight, washed, and resuspended in 0.5% BSA in HEPES buffer. For inhibitor assays, mast cells were then preincubated for 1 hour at 37°C with or without inhibitors; JNJ-7777120 (10 μM, ab144405, Abcam, ON, Canada) or TAK-242 (10 μM, 13871, Cayman Chemical, Burlington, Canada). The BMMC (3 x 10^5^ cells/100 μL) were added to the upper chamber of the Transwell^®^, and incubated for 20 minutes. The inserts were then transferred to a fresh well containing 10% cecal bacteria supernatant in 0.5% BSA in HEPES buffer, and the plate was incubated for 4 hours at 37°C. Migrated cells were collected and counted using microscopy.

*Colonic and BMMC gene expression*

BMMC (5.6 x 10^6^ cells/mL), co-cultured with 10% bacterial supernatant for 4 hours at 37°C, were collected and centrifuged (1300 rmp). The supernatants were collected and stored at -80°C for ELISA. Freshly collected colon tissues were stored in RNAlater (Sigma) at -80°C. Cell pellets and colon tissues were dissolved or homogenized with RLT buffer (Qiagen, Toronto, Canada) containing 1% β-mercaptoethanol. Total RNA extractions were conducted with the RNeasy Mini Kit (Qiagen, Toronto, Canada) according to the manufacturer’s instructions. DNase digestion during purification was carried out using the RNase-free DNase (Qiagen). The RNA was transcribed to cDNA with SuperScript™ III Reverse Transcriptase (Invitrogen, Burlington, ON, Canada) according to the manufacturer’s instructions. The mRNA expression of mouse intestinal H_1_R, H_2_R, H_4_R, TLR2, TLR4 and CXCL12 were analyzed by real time qPCR. One μg of the total RNA was transcribed to cDNA with SuperScript™ III Reverse Transcriptase (Invitrogen, Burlington, ON, Canada) according to the manufacturer’s instructions. The cDNA was amplified with Sso Fast^TM^ EvaGreen® Supermix (Bio-Rad and Applied Biosystems, Mississauga, ON, Canada) using a CFX Connect Real-Time System (Bio-Rad and Applied Biosystems). H_2_R, H_4_R and CXCL12 primers were from Qiagen Quantitect, and H_1_R, TLR2 and TLR4 primers were from Bio-Rad and Applied Biosystems. GAPDH (Mobix Laboratory, McMaster University, Hamilton, Canada) and β-actin (Bio-Rad and Applied Biosystems) were used as reference genes to normalize the data.

*Histamine ELISA*

Histamine was measured with the Mouse Histamine ELISA Kit (LS-F28398, LifeSpan Biosciences, Burlington, Canada), according to manufacturer’s instructions.

*Statistical Analysis*

The data are presented as median (IQD) or mean ± SEM. The data were analyzed by Kruskal-Wallis test followed by Dunn's post-test or Mann-Whitney test. Associations between tryptase positive cells and microbial genera were analysed with the Spearman’s rank correlation test. The resulting P values were were corrected for multiple comparisons, allowing 5% of False Discovery Rate. P<0.05 was considered statistically significant.

**References**

1. De Palma G, Lynch MD, Lu J, Dang VT, Deng Y, Jury J, et al. Transplantation of fecal microbiota from patients with irritable bowel syndrome alters gut function and behavior in recipient mice. Sci Transl Med 2017; 9.

2. Whelan FJ, Surette MG. A comprehensive evaluation of the sl1p pipeline for 16S rRNA gene sequencing analysis. Microbiome 2017; 5:100.

3. Bartram AK, Lynch MD, Stearns JC, Moreno-Hagelsieb G, Neufeld JD. Generation of multimillion-sequence 16S rRNA gene libraries from complex microbial communities by assembling paired-end illumina reads. Appl Environ Microbiol 2011; 77:3846-52.

4. Callahan BJ, McMurdie PJ, Rosen MJ, Han AW, Johnson AJA, Holmes SP. DADA2: High-resolution sample inference from Illumina amplicon data. Nature Methods 2016; 13:581-+.

5. Bolyen E, Rideout JR, Dillon MR, Bokulich N, Abnet CC, Al-Ghalith GA, et al. Reproducible, interactive, scalable and extensible microbiome data science using QIIME 2. Nature Biotechnology 2019; 37:852-7.

6. Wang Q, Garrity GM, Tiedje JM, Cole JR. Naive Bayesian classifier for rapid assignment of rRNA sequences into the new bacterial taxonomy. Appl Environ Microbiol 2007; 73:5261-7.

7. Quast C, Pruesse E, Yilmaz P, Gerken J, Schweer T, Yarza P, et al. The SILVA ribosomal RNA gene database project: improved data processing and web-based tools. Nucleic Acids Res 2013; 41:D590-6.

8. Yilmaz P, Parfrey LW, Yarza P, Gerken J, Pruesse E, Quast C, et al. The SILVA and "All-species Living Tree Project (LTP)" taxonomic frameworks. Nucleic Acids Res 2014; 42:D643-8.

9. Morgan XC, Kabakchiev B, Waldron L, Tyler AD, Tickle TL, Milgrom R, et al. Associations between host gene expression, the mucosal microbiome, and clinical outcome in the pelvic pouch of patients with inflammatory bowel disease. Genome Biol 2015; 16:67.

10. McMurdie PJ, Holmes S. phyloseq: An R Package for Reproducible Interactive Analysis and Graphics of Microbiome Census Data. Plos One 2013; 8.

11. Shimbori C, Upagupta C, Bellaye PS, Ayaub EA, Sato S, Yanagihara T, et al. Mechanical stress-induced mast cell degranulation activates TGF-beta1 signalling pathway in pulmonary fibrosis. Thorax 2019; 74:455-65.

12. Khambati I, Han S, Pijnenburg D, Jang H, Forsythe P. The bacterial quorum-sensing molecule, N-3-oxo-dodecanoyl-L-homoserine lactone, inhibits mediator release and chemotaxis of murine mast cells. Inflamm Res 2017; 66:259-68.

**
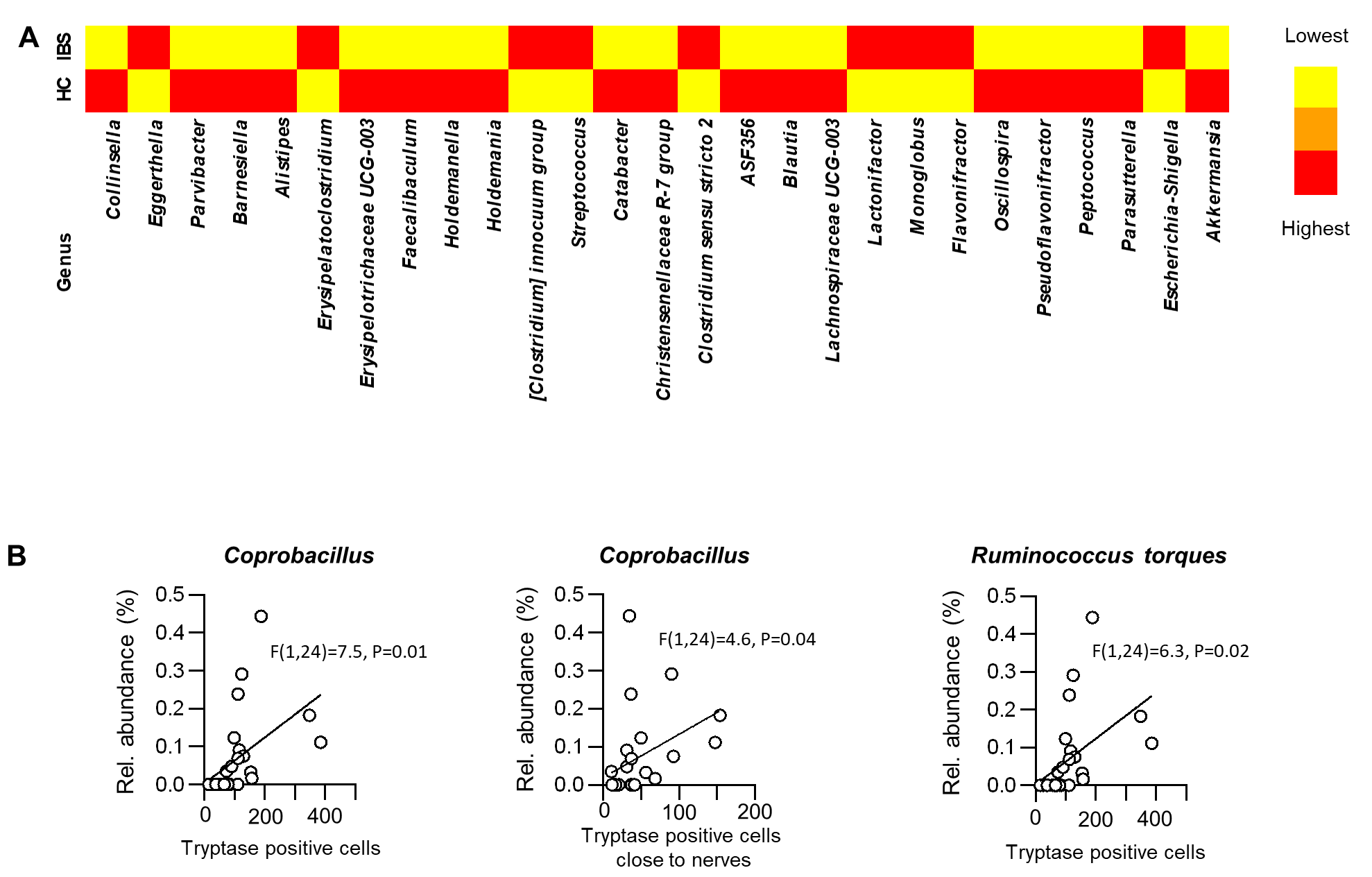
**

**Supplementary Figure 1*:* Gut microbiota composition differs between HC and IBS**

**mice**

**A:** Heatmap of the genera that differed between HC and IBS colonized mice when testing with MAasLin. Each taxon was represented individually. Coloring of the heatmap was proportional to the relative abundance of each taxon in the sample and relative to the neighboring group (HC vs IBS; the higher abundance would be darker than the lower abundance). Each row represents the average relative abundance for each group, HC and IBS colonized mice.

**B:** Correlations of bacteria with mast cells or mast cells colocalized with neural fibers. The data was analysed with Spearman test and with simple linear regression. The linear regression equation is reported on each panel.
